# Supplementary material for: Development and internal validation of a field-based triage tool for lumbopelvic-hip dysfunction in collegiate athletes
Source: PLoS One. 2026 Sep 9;21(9):e0348820. doi: 10.1371/journal.pone.0348820 (PMC13557378; doi:10.1371/journal.pone.0348820)
Supplement: Supplementary file 1 [file pone.0348820.s005.pdf]

ScreenIT Report

This is an automated report for 10.64898+2026.04.23.26351566.pdf. This report was solicited by the PLOS One editorial team and provided by ScreenIT.

ScreenIT is an independent group of scientists developing automated tools that analyze academic papers. A set of automated tools screened your submitted manuscript and provided the report below. Each tool was created by your academic colleagues with the goal of helping authors.

The tools look for factors that are important for transparency, rigor and reproducibility, and we hope that the report might help you to improve reporting in your manuscript. Within the report you will find links to more information about the items that the tools check. These links include helpful papers, websites, or videos that explain why the item is important.

While our screening tools aim to improve and maintain quality standards they may, on occasion, miss nuances specific to your study type or flag something incorrectly. Each tool has limitations that are described on the ScreenIT website. The tools screen the main file for the paper; they are not able to screen supplements stored in separate files.

Please note that the Academic Editor had access to these comments while making a decision on your manuscript. The Academic Editor may ask that issues flagged in this report be addressed.

If you would like to provide feedback on the ScreenIT tool, please email the team at ScreenIt@bih-charite.de. If you have questions or concerns about the review process, please contact the PLOS One office at plosone@plos.org.

How to read the report:

- no action required
- review for potential issues
- a critical issue was found by a tool
- may not be applicable to your paper

- more information about this item can be obtained using this link

Rigor

✓	Ethics	Consent: "All participants provided written informed consent, and the study was approved by the Kaohsiung Medical University Institutional Review Board (KMUHIRB-F(II)20210113, 2021/6/29-2026/8/31) in accordance with the Declaration of Helsinki."<br>IRB: "All participants provided written informed consent, and the study was approved by the Kaohsiung Medical University Institutional Review Board (KMUHIRB-F(II)20210113, 2021/6/29-2026/8/31) in accordance with the Declaration of Helsinki."
✓	Inclusion and Exclusion Criteria	"Inclusion criteria were: (1) active roster status on a varsity team; (2) age 1830 years; and (3) a minimum training volume of 10 hours per week."
✓	Flow charts and attrition	Thank you for including a study flow chart and information about attrition and exclusions to help readers evaluate the risk of bias.<br><br>Sentence about attrition: not detected. Please provide information about the drop-out of subjects, or loss of animals or samples. This could be done using a study flow chart, or described in the text.
✓	Sex as a biological variable	"Data included sex (male/female), age (years), height (cm), weight (kg), body mass index (BMI, kg/m), years of sport participation, and average weekly training volume (hours/week)."
✓	Subject Demographics	Age: "Inclusion criteria were: (1) active roster status on a varsity team; (2) age 1830 years; and (3) a minimum training volume of 10 hours per week."
✓	Randomization	"In Phase 2, inter-rater reliability of the same predefined criteria was evaluated in a randomly selected subset of athletes (n = 41)."
✓	Blinding	"Examiners performing the physical assessments were blinded to participants demographic information, injury history, and questionnaire responses at the time of testing."

?	Power Analysis	Not detected. Please state how you determined the sample size for the study. If you performed a power or sample size calculation, please provide details.
n/a	Replication	not required.

Generated by Barzooka (flow charts), SciScore (everything else).
SciScore only screens the text of the methods section; we apologize if the tool has missed items reported elsewhere in the paper or supplements.

Resources

Persistent unique identifiers for research resources, RRIDs, can be used to track these key resources though the literature. They are requested or required in many journals, but even if your journal does not require them, we strongly encourage the use of these for the readers of papers who wish to easily track down the key materials like antibodies, software tools, or mice.

Software and Algorithms	"All statistical analyses were performed using SPSS version 25.0 (IBM Corp., Armonk, NY, USA) with statistical significance set at $p < 0.05$."	? SPSS suggested: SPSS, RRID:SCR_002865. Please make sure this RRID suggestion is accurate before including in your manuscript.
Software and Algorithms	"DCA was performed in Python 3.11.3."	? Python suggested: python-bx (RRID:SCR_024202. Please make sure this RRID suggestion is accurate before including in your manuscript.

Generated by SciScore.
SciScore only screens the text of the methods section; we apologize if the tool has missed items reported elsewhere in the paper or supplements.

Transparency

?	Open code	If you wrote code to analyze your data, please consider sharing the code in a public repository. This makes it easier for others to reproduce your analyses, and may also aid others seeking to analyze similar datasets.

?	Open data	ℹ If permitted, sharing data on a public repository with a persistent identifier (a DOI or accession number) can improve reproducibility and make it easier for other scientists to expand on your work. Papers with open data are cited more often than papers without open data. Some institutions have an expert who can provide advice on data sharing.
✓	Self-acknowledged limitations	ℹ "Thus, these negative weights may have helped reduce over-classification of athletes with non-specific flexibility limitations. However, any athlete demonstrating overt neurological deficits or progressive redflag symptoms should undergo comprehensive medical evaluation. An important strength of CTSS is its capacity to identify functional abnormalities in athletes who may not report prominent symptoms, thereby addressing a recognized limitation of symptom-based triage approaches (4). DCA further contextualized the clinical utility of the model (Figure 2D). Compared with treat-all and treat-none strategies, CTSS demonstrated consistently higher net benefit across the prespecified threshold probability range of 20%50%. At the 50% threshold probability, corresponding to the operational score threshold of 9, the net benefit was 0.319, suggesting improved decision balance between identifying athletes requiring priority intervention and reducing unnecessary referrals. These findings suggest t..."
✓	Conflict of interest statement	ℹ Thank you for stating any conflicts of interest.
✓	Funding statement	ℹ Thank you for reporting your funding source(s), or stating that there was no specific funding for this work.
n/a	Registration statement	ℹ A registered protocol makes the planned research project more transparent and can protect against bias. Please consider registering future confirmatory studies.

Generated by ODDPub, limitation-recognizer, rtransparent, trial-identifier.

Figures

	No bar graphs of	ℹ Thank you for not using bar graphs to present continuous data. Many different datasets and data

✓	continuous data	distributions can lead to the same bar graph, and the actual data may suggest different conclusions from the summary statistics alone.
✓	No rainbow color maps	ⓘ Thank you for not using rainbow colormaps. Rainbow color maps distort readers' perception of the data by introducing visual artifacts and are not accessible to colorblind readers.

Generated by JetFighter, Barzooka.
