# Supplementary material for: Development and internal validation of a field-based triage tool for lumbopelvic-hip dysfunction in collegiate athletes
Source: PLoS One. 2026 Sep 9;21(9):e0348820. doi: 10.1371/journal.pone.0348820 (PMC13557378; doi:10.1371/journal.pone.0348820)
Supplement: Supplementary file 2 [file pone.0348820.s002.docx]

**Table S1.** Clinical Assessment Protocols for Lumbopelvic-hip Screening

| Functional Domain | Test Item | Screening Objective | Positive Criteria |
| --- | --- | --- | --- |
| A. Neural Sensitivity | Lasegue’s straight-leg raise (SLR) | Neural tension / lumbar radiculopathy | Reproduction of radicular symptoms below ~70° of hip flexion. |
|  | Double-leg raise test | Lumbar instability | Pain provoked at a lower angle compared to SLR or inability to maintain controlled lift. |
|  | Bonnet’s sign | Piriformis involvement / deep gluteal sciatic entrapment | Deep gluteal pain or radiating sciatic symptoms with passive internal rotation/adduction. |
| B. Articular Loading & Sensitivity | Advancement test | Sacroiliac (SI) joint dysfunction | Asymmetry or reproduction of pain during forward flexion. |
|  | Belt test | Lumbar vs. SI pain differentiation | Pain persists with pelvic stabilization (lumbar origin) or decreases with stabilization (SI origin). |
|  | FABER (Patrick’s) test | Hip or SI joint pathology | Pain in the groin/hip (hip pathology) or gluteal/SI region (SI involvement). |
|  | Internal/external hip rotation test | Hip joint mobility / impingement | Restricted ROM, pain, or side-to-side asymmetry. |
|  | Gaenslen’s test | SI joint stress | SI region pain during torsional stress in hip extension/flexion position. |
| C. Myofascial Length | Thomas test | Hip flexor tightness | Inability to maintain hip extension; contralateral hip flexion indicates iliopsoas tightness. |
|  | Ely’s sign | Rectus femoris tightness | Passive knee flexion induces ipsilateral hip flexion. |
|  | Hyperextension test | Lumbar facet loading | Localized low-back pain reproduced during lumbar extension. |
|  | Single-leg hip extension test | Functional lumbar–hip-pelvic control | Reproduction of low-back/gluteal pain or inability to stabilize pelvis during active hip extension. |
|  | Ober’s test | Iliotibial band tightness | Inability of thigh to adduct past midline when tested passively. |
| Note. SLR = straight-leg raise; SI = sacroiliac; ROM = range of motion; PSIS = posterior superior iliac spine. All tests were performed according to standard clinical examination protocols. | | | |
