# Supplementary material for: Development and internal validation of a field-based triage tool for lumbopelvic-hip dysfunction in collegiate athletes
Source: PLoS One. 2026 Sep 9;21(9):e0348820. doi: 10.1371/journal.pone.0348820 (PMC13557378; doi:10.1371/journal.pone.0348820)
Supplement: Supplementary file 3 [file pone.0348820.s003.docx]

**Table S2.** Bootstrap Validation of Logistic Regression Coefficients (1,000 Resamples)

| Predictors | β coefficient | Bootstrap SE | p-value | BCa 95% CI |
| --- | --- | --- | --- | --- |
| Bonnet’s sign (positive) | 0.668 | 0.183 | 0.001 | 0.300 - 1.053 |
| Internal/external hip rotation test (positive) | 0.544 | 0.204 | 0.004 | 0.113 - 0.996 |
| Thomas test (positive) | 0.667 | 0.285 | 0.011 | 0.091 - 1.320 |
| Hyperextension test (positive) | 0.849 | 0.411 | 0.001 | 0.471 - 1.328 |
| LBP on day of evaluation | 0.865 | 0.227 | 0.001 | 0.413 - 1.304 |
| LBP in last 6 months | 0.885 | 0.210 | 0.001 | 0.424 - 1.338 |
| Pain-now at spinal process (L1–L5) | 0.943 | 0.361 | 0.005 | 0.260 - 1.763 |
| Pain-now at right paraspinal region | 0.753 | 0.309 | 0.0.014 | 0.197 - 1.531 |
| Pain-now at left gluteal region | 0.845 | 0.408 | 0.028 | 0.047 - 1.879 |
| Gluteus medius weakness | 0.408 | 0.172 | 0.020 | 0.043 - 0.758 |
| Piriformis weakness | 0.558 | 0.163 | 0.009 | 0.128 - 0.993 |
| Body Mass Index (per kg/m²) | 0.059 | 0.023 | 0.003 | 0.014 - 0.106 |
| Lasegue’s straight-leg raise (positive) | -0.962 | 0.703 | 0.143 | -2.300 - 0.365 |
| Hamstring muscle weakness | -0.453 | 0.257 | 0.077 | -0.959 - 0.024 |
| Note. Estimates based on 1,000 bootstrap resamples. BCa = bias-corrected and accelerated confidence interval; SE = standard error. variables are ordered according to the final weighted scoring system. | | | | |
