# Supplementary material for: Development and internal validation of a field-based triage tool for lumbopelvic-hip dysfunction in collegiate athletes
Source: PLoS One. 2026 Sep 9;21(9):e0348820. doi: 10.1371/journal.pone.0348820 (PMC13557378; doi:10.1371/journal.pone.0348820)
Supplement: Supplementary file 4 [file pone.0348820.s004.docx]

**Table S3.** Overlap between candidate predictor categories, expert outcome adjudication, and variables retained in the final multivariable model.

| Candidate predictor category | Number of variables | Use in expert outcome adjudication | Variables Retained in Final Model (Table 2) |
| --- | --- | --- | --- |
| Demographic characteristics (age, gender, BMI, sport experience and training hours) | 5 | Not considered during expert outcome adjudication. | Only BMI was retained; age, sex, sport experience, and training hours were excluded. |
| LBP prevalence, regional injury history, and LPH pain location | 18 | Considered together with other clinical findings. | LBP on the day of evaluation, LBP in the last 6 months, and only 3 of the 10 pain-location variables were retained. |
| Lower back and hip ROM | 10 | Considered as part of the overall functional assessment. | None of the ROM variables remained independent predictors after multivariable adjustment. |
| Bilateral MMT | 7 | Considered together with other functional findings. | Only gluteus medius, piriformis, and hamstring muscle weakness were retained. |
| Orthopedic provocation tests | 13 | Considered as part of the integrated clinical assessment. | Only five of the 13 orthopedic provocation tests (Bonnet's sign, internal/external hip rotation test, Thomas test, hyperextension test, and Lasegue's straight leg test) were retained. |
| Note: Candidate predictors were derived from the same standardized assessment. Expert outcome adjudication integrated multiple clinical findings rather than relying on any single variable. LBP, low back pain; LPH, lumbopelvic-hip; ROM, range of motion; MMT, manual muscle testing | | | |
