# Supplementary material for: Development and internal validation of a field-based triage tool for lumbopelvic-hip dysfunction in collegiate athletes
Source: PLoS One. 2026 Sep 9;21(9):e0348820. doi: 10.1371/journal.pone.0348820 (PMC13557378; doi:10.1371/journal.pone.0348820)
Supplement: Supplementary file 5 [file pone.0348820.s006.docx]

**Reviewer Report**

**Manuscript ID:** PONE-D-26-15749

**Title:** *Development and Internal Validation of a Field-Based Triage Tool for Lumbopelvic-Hip Dysfunction in Collegiate Athletes*

**Article Type:** Research Article

A) **Major Revision Required:-**

The manuscript addresses an important and clinically relevant topic in sports medicine by developing a field-based Clinical Triage Scoring System (CTSS) for identifying collegiate athletes requiring priority intervention for lumbopelvic-hip dysfunction. The study includes a relatively large sample (n = 864), systematic assessment procedures, and internal validation using bootstrap methods. The reported discrimination (AUROC = 0.851) and calibration appear promising.

However, several substantial methodological and reporting concerns limit confidence in the current findings. The most important issue is the potential circularity between the predictor variables and the expert-derived reference standard, which may have inflated model performance. Additional concerns relate to predictor selection strategy, outcome definition, lack of external validation, and insufficient adherence to contemporary prediction-model reporting standards (TRIPOD/TRIPOD-AI).

The manuscript has merit but requires substantial revision before it can be considered for publication.

**Major Comments**

**1. Potential Circularity and Incorporation Bias**

The most critical limitation concerns the development of the reference standard.

The outcome ("priority intervention") was derived from expert consensus using many of the same clinical findings that were subsequently entered as predictors in the logistic regression model. Although the authors acknowledge this limitation, the degree of overlap appears substantial.

Examples include:

- Pain location
- Low back pain history
- Muscle weakness
- Orthopedic provocation tests
- Functional examination findings

Consequently, the model may largely reproduce expert decision-making rather than independently predict clinically meaningful need for intervention.

**Recommendation**

The authors should:

- Explicitly identify which variables were used in the expert classification process.
- Provide a table showing overlap between predictor candidates and outcome criteria.
- Quantify the risk of incorporation bias.
- Clarify whether any predictors were excluded from outcome adjudication.

Without this clarification, the reported AUROC may be artificially inflated.

**2. Outcome Definition Requires Stronger Justification**

The manuscript develops a tool to predict "priority intervention."

However:

- This outcome is not an objective clinical endpoint.
- It is not linked to future injury.
- It is not linked to rehabilitation success.
- It is not linked to athlete performance outcomes.

Instead, it reflects expert opinion regarding clinical priority.

This limits interpretability and clinical applicability.

**3. Use of Stepwise Logistic Regression**

The authors employed forward stepwise logistic regression.

Stepwise approaches are increasingly discouraged in prediction-model development because they:

- Increase over fitting risk.
- Produce unstable coefficients.
- Inflate apparent performance.
- Yield poor reproducibility across datasets.

Even though bootstrap validation was performed, the limitation remains substantial.

**4. Lack of External Validation**

The study performs only internal validation.

Prediction models frequently demonstrate reduced performance when tested in new populations.

The authors correctly acknowledge this limitation, but stronger emphasis is needed.

- External validation is mandatory before clinical adoption.
- Generalizability remains unknown.

**5. Missing Assessment of Inter-Rater Reliability for CTSS Components**

Although expert-panel reliability was reported (κ = 0.977), the study does not evaluate reliability of the actual CTSS assessment items.

**7. Data Availability Statement Appears Inconsistent**

The submission states:

"All relevant data are within the manuscript and its Supporting Information files."

However:

- Raw data are not presented.
- Model-development datasets are not available.
- Bootstrap validation outputs are not available.

**Recommendation**

The authors should deposit:

- De-identified dataset
- Statistical code
- Model coefficients
- Validation scripts in a public repository consistent with PLOS ONE data-sharing requirements.

**B) Minor Comments**

**1. Terminology**

The manuscript alternates between:

- Low back pain (LBP)
- Lumbopelvic-hip dysfunction (LPH dysfunction)

These concepts are related but not identical.

**2. Reporting Standard**

Although STROBE is cited, prediction-model studies should primarily follow:

- TRIPOD reporting guidelines

A completed TRIPOD checklist should be included.

**3. Participant Sex Distribution**

The sample is:

- 80.8% male

This substantially limits applicability to female athletes.

The discussion should emphasize this limitation more strongly.

**4. Clarify Missing Data Handling**

The manuscript reports:

- 61 participants excluded due to missing data

The authors should provide:

- Pattern of missingness
- Variables affected
- Comparison between included and excluded participants

**5. Figure Presentation**

Figure 2 should include:

- Calibration intercept
- Calibration slope
- Confidence intervals around calibration curve to improve interpretability.

**Strengths of the Manuscript**

1. Large sample size (n = 864).
2. Multi-centre recruitment from 10 universities.
3. Clinically relevant sports medicine problem.
4. Detailed assessment procedures.
5. Bootstrap internal validation.
6. Calibration and decision curve analysis included.
7. Practical field-based focus with potential clinical applicability.
