# Supplementary material for: Development and internal validation of a field-based triage tool for lumbopelvic-hip dysfunction in collegiate athletes
Source: PLoS One. 2026 Sep 9;21(9):e0348820. doi: 10.1371/journal.pone.0348820 (PMC13557378; doi:10.1371/journal.pone.0348820)
Supplement: Supplementary file 6 [file pone.0348820.s008.doc]

**Response to Review Comments**

Manuscript ID: PONE-D-26-15749

**Reply to Reviewer.**

| Reviewer#1: Response to the comment on ScreenIT | Authors' Response |
| --- | --- |
| Power Analysis:  Not detected. Please state how you determined the sample size for the study. If you performed a power or sample size calculation, please provide details. | Power analysis was not performed because this study aimed to develop a prediction model rather than test a statistical hypothesis. Instead, the adequacy of the sample size was assessed using the Events per Variable (EPV) approach, which is considered more appropriate for prediction model development. Please refer to Page 12 for details. |
| Software and Algorithms   - SPSS suggested: SPSS, RRID:SCR_002865. Please make sure this RRID suggestion is accurate before including in your manuscript. - Python suggested: python-bx (RRID:SCR_024202. Please make sure this RRID suggestion is accurate before including in your manuscript. | The RRIDs for SPSS (IBM Corp., v25.0; RRID:SCR_002865) and Python (v3.11.3; used for calibration slope calculation and Decision Curve Analysis; RRID:SCR_008394, Python Programming Language) have been added. Although SciScore initially suggested RRID:SCR_024202 (python-bx), we confirmed via SciCrunch (scicrunch.org) that SCR_008394 is the correct identifier. |

| Reviewer#2 Comments: | Authors' Response |
| --- | --- |
| The manuscript addresses an important and clinically relevant topic in sports medicine by developing a field-based Clinical Triage Scoring System (CTSS) for identifying collegiate athletes requiring priority intervention for lumbopelvic-hip dysfunction. The study includes a relatively large sample (n = 864), systematic assessment procedures, and internal validation using bootstrap methods. The reported discrimination (AUROC = 0.851) and calibration appear promising.  However, several substantial methodological and reporting concerns limit confidence in the current findings. The most important issue is the potential circularity between the predictor variables and the expert-derived reference standard, which may have inflated model performance. Additional concerns relate to predictor selection strategy, outcome definition, lack of external validation, and insufficient adherence to contemporary prediction-model reporting standards (TRIPOD/TRIPOD-AI).  The manuscript has merit but requires substantial revision before it can be considered for publication. | We sincerely thank the reviewer for the constructive and insightful comments. We appreciate the positive evaluation of the clinical relevance, sample size, and model performance. We have carefully revised the manuscript to address all methodological and reporting concerns. Our point-by-point responses are provided below. |
| **Major comments** |  |
| 1. Potential Circularity and Incorporation Bias  The most critical limitation concerns the development of the reference standard.  The outcome ("priority intervention") was derived from expert consensus using many of the same clinical findings that were subsequently entered as predictors in the logistic regression model. Although the authors acknowledge this limitation, the degree of overlap appears substantial.  Examples include:  • Pain location  • Low back pain history  • Muscle weakness  • Orthopedic provocation tests  • Functional examination findings  Consequently, the model may largely reproduce expert decision-making rather than independently predict clinically meaningful need for intervention.  Recommendation  The authors should:  • Explicitly identify which variables were used in the expert classification process.  • Provide a table showing overlap between predictor candidates and outcome criteria.  • Quantify the risk of incorporation bias.  • Clarify whether any predictors were excluded from outcome adjudication.  Without this clarification, the reported AUROC may be artificially inflated. | Thank you for this important comment. We agree that incorporation bias cannot be completely excluded because both the expert-derived reference standard and the candidate predictors were derived from the same standardized clinical assessment.  To address this concern, we have:   - Expanded the Methods to clarify the expert outcome adjudication process. - Added Table S3 to explicitly summarize the overlap between candidate predictor categories, expert outcome adjudication, and variables retained in the final multivariable model. - Quantified the extent of overlap. Of the 53 candidate predictors, 48 (90.6%) were considered during expert outcome adjudication, whereas only 14 (26.4%) were retained in the final multivariable model, indicating that only a subset of variables considered during expert adjudication contributed independently to the final model   Revised the Discussion and Limitations to acknowledge the potential for incorporation bias and the possibility that model discrimination may be optimistic.  Revisions incorporated: Page 8, 18, and 19 |
| 2. Outcome Definition Requires Stronger Justification  The manuscript develops a tool to predict "priority intervention." However:  • This outcome is not an objective clinical endpoint.  • It is not linked to future injury.  • It is not linked to rehabilitation success.  • It is not linked to athlete performance outcomes.  Instead, it reflects expert opinion regarding clinical priority.  This limits interpretability and clinical applicability. | We have clarified that the predefined outcome represents athletes requiring further clinical assessment and management according to predefined expert consensus framework, rather than an objective pathological diagnosis or prognostic endpoint.  Revisions incorporated: Page 8 and 19 |
| 3. Use of Stepwise Logistic Regression  The authors employed forward stepwise logistic regression.  Stepwise approaches are increasingly discouraged in prediction-model development because they:  • Increase over fitting risk.  • Produce unstable coefficients.  • Inflate apparent performance.  • Yield poor reproducibility across datasets.  Even though bootstrap validation was performed, the limitation remains substantial. | We thank the reviewer for this important comment. We acknowledge the recognized limitations of stepwise logistic regression, including potential instability in variable selection, coefficient estimation, and reproducibility across different datasets. This approach was selected to derive a parsimonious and clinically interpretable field-based scoring system. To mitigate these limitations, candidate predictors were prespecified based on clinical reasoning, the events-per-variable ratio was favorable (EPV = 33.1), and internal validation was performed using 1,000 bootstrap resamples. We have revised the Discussion and Limitations to explicitly acknowledge these methodological limitations and emphasize that bootstrap validation cannot substitute for external validation. Accordingly, independent validation in diverse athletic populations is required to confirm model stability, reproducibility, and generalizability before broader clinical implementation.  Revisions incorporated: Pages 18 and 20. |
| 4. Lack of External Validation  The study performs only internal validation.  Prediction models frequently demonstrate reduced performance when tested in new populations.  The authors correctly acknowledge this limitation, but stronger emphasis is needed.  • External validation is mandatory before clinical adoption.  • Generalizability remains unknown. | We strengthened the Limitations to emphasize that external validation is required before broader clinical implementation and that the model's generalizability remains to be established.  Revisions incorporated: Page 19 |
| 5. Missing Assessment of Inter-Rater Reliability for CTSS Components  Although expert-panel reliability was reported (κ = 0.977), the study does not evaluate reliability of the actual CTSS assessment items. | We thank the reviewer for this important observation. Our reported Fleiss’ weighted kappa reflects agreement for the expert-assigned four-level triage classifications rather than the reliability of individual CTSS assessment items. This is because, rather than relying on item-by-item score comparisons or any single abnormal finding, our expert framework incorporated integrated clinical judgment across multiple domains. We have revised the Methods to explicitly clarify this distinction and avoid potential misunderstanding.  Revisions incorporated: Page 9 |
| 6. Data Availability Statement Appears Inconsistent  The submission states:  "All relevant data are within the manuscript and its Supporting Information files."  However:  • Raw data are not presented.  • Model-development datasets are not available.  • Bootstrap validation outputs are not available.  Recommendation  The authors should deposit:  • De-identified dataset  • Statistical code  • Model coefficients  • Validation scripts in a public repository consistent with PLOS ONE data-sharing requirements. | We thank the reviewer for this helpful comment. To ensure compliance with the PLOS ONE Data Availability Policy, we have now provided the requested materials as Supporting Information. All materials—including the de-identified dataset, model coefficients (including bootstrap validation outputs), and statistical code—have been consolidated into a single compressed archive and uploaded as a “Supporting Information – Compressed/ZIP File Archive.”  Revisions: Please refer to the Supporting Information for the revised materials. |
| Minor Comments |  |
| 1. Terminology  The manuscript alternates between:  • Low back pain (LBP)  • Lumbopelvic-hip dysfunction (LPH dysfunction)  These concepts are related but not identical. | We thank the reviewer for this helpful comment. We agree that low back pain (LBP) and lumbopelvic–hip (LPH) dysfunction are related but distinct concepts. The manuscript has been revised to consistently distinguish LBP from LPH dysfunction and to clarify that the LPH functional assessment was used to identify functional impairments associated with LBP for field-based clinical triage, rather than to diagnose LBP itself. These clarifications have been incorporated throughout the Abstract, Introduction, Methods, and Discussion.  Revisions incorporated: Page 2, 3, 8, and 19 |
| 2. Reporting Standard  Although STROBE is cited, prediction-model studies should primarily follow:  • TRIPOD reporting guidelines  A completed TRIPOD checklist should be included. | We thank the reviewer for this helpful suggestion. The Methods section has been revised to indicate that the study was also reported in accordance with the TRIPOD guidelines. In addition, a completed TRIPOD checklist has been included as Supporting Information.  Revisions incorporated: Page 4 and TRIPOD Checklist (Supporting Information). |
| 3. Participant Sex Distribution  The sample is:  • 80.8% male  This substantially limits applicability to female athletes.  The discussion should emphasize this limitation more strongly. | We thank the reviewer for this helpful comment. We have strengthened the Limitations section to emphasize that the predominantly male cohort may limit the generalizability of the CTSS, particularly to female athletes. We also explicitly state that validation in independent and diverse athletic populations, including female athletes, is required before broader clinical implementation.  Revisions incorporated: Page 19 |
| 4. Clarify Missing Data Handling  The manuscript reports:  • 61 participants excluded due to missing data  The authors should provide:  • Pattern of missingness  • Variables affected  • Comparison between included and excluded participants | We thank the reviewer for this important comment. A prespecified complete-case analysis was performed, and participants with incomplete data were excluded. Most missing data resulted from incomplete completion of the standardized on-site assessment protocol (e.g., scheduling or time constraints) rather than missing individual variables. We have clarified this in the Methods. Given the small proportion of excluded participants (61/945, 6.5%), formal comparisons between included and excluded participants were not performed. |
| 5. Figure Presentation  Figure 2 should include:  • Calibration intercept  • Calibration slope  • Confidence intervals around calibration curve to improve interpretability. | We thank the reviewer for this helpful suggestion. Figure 2 has been revised to include the calibration intercept, calibration slope, and the 95% confidence band around the calibration curve. Corresponding descriptions have also been incorporated into the Methods and Results.  Revisions incorporated: Figure 2 and Page 13 |

| Reviewer#3 Comments: | Authors' Response |
| --- | --- |
| The manuscript entitled “Development and Internal Validation of a Field-Based Triage Tool for Lumbopelvic-Hip Dysfunction in Collegiate Athletes” addresses an important topic in sports medicine and rehabilitation. The development of a practical field-based clinical triage scoring system (CTSS) for identifying athletes requiring priority intervention is relevant and may have potential clinical utility. The study includes a relatively large sample size and employs several statistical techniques, including logistic regression, ROC analysis, calibration assessment, bootstrap validation, and decision curve analysis. The manuscript has several strengths, including a clear study objective, adequate sample size, and a clinically applicable scoring system derived from routine assessments. The reported discrimination performance (AUROC = 0.851) and calibration results indicate promising model performance. | We sincerely thank the reviewer for the positive evaluation of our manuscript. |
