# Supplementary material for: Development and internal validation of a field-based triage tool for lumbopelvic-hip dysfunction in collegiate athletes
Source: PLoS One. 2026 Sep 9;21(9):e0348820. doi: 10.1371/journal.pone.0348820 (PMC13557378; doi:10.1371/journal.pone.0348820)
Supplement: Peer review history

# Peer review: Decision Letter 0

**DOI:** 10.1371/journal.pone.0348820.r001

**Authors:** Aliah Shaheen

25 Jun 2026

Dear Dr. Wu,

Thank you for submitting your manuscript to PLOS ONE. After careful consideration, we feel that it has merit but does not fully meet PLOS ONE’s publication criteria as it currently stands. Therefore, we invite you to submit a revised version of the manuscript that addresses the points raised during the review process.

Please submit your revised manuscript by Aug 09 2026 11:59PM. If you will need more time than this to complete your revisions, please reply to this message or contact the journal office at plosone@plos.org. When you're ready to submit your revision, log on to https://www.editorialmanager.com/pone/ and select the 'Submissions Needing Revision' folder to locate your manuscript file.

- A letter that responds to each point raised by the academic editor and reviewer(s). You should upload this letter as a separate file labeled 'Response to Reviewers'.
- A marked-up copy of your manuscript that highlights changes made to the original version. You should upload this as a separate file labeled 'Revised Manuscript with Track Changes'.
- An unmarked version of your revised paper without tracked changes. You should upload this as a separate file labeled 'Manuscript'.

If you would like to make changes to your financial disclosure, please include your updated statement in your cover letter. Guidelines for resubmitting your figure files are available below the reviewer comments at the end of this letter.

If applicable, we recommend that you deposit your laboratory protocols in protocols.io to enhance the reproducibility of your results. Protocols.io assigns your protocol its own identifier (DOI) so that it can be cited independently in the future. For instructions see: https://journals.plos.org/plosone/s/submission-guidelines#loc-laboratory-protocols. Additionally, PLOS ONE offers an option for publishing peer-reviewed Lab Protocol articles, which describe protocols hosted on protocols.io. Read more information on sharing protocols at https://plos.org/protocols?utm_medium=editorial-email&utm_source=authorletters&utm_campaign=protocols.

As the corresponding author, your ORCID iD is verified in the submission system and will appear in the published article. PLOS supports the use of ORCID, and we encourage all coauthors to register for an ORCID iD and use it as well. Please encourage your coauthors to verify their ORCID iD within the submission system before final acceptance, as unverified ORCID iDs will not appear in the published article. *Only*  the individual author can complete the verification step; PLOS staff *cannot*  verify ORCID iDs on behalf of authors.

We look forward to receiving your revised manuscript.

Kind regards,

Aliah Faisal Shaheen

Academic Editor

PLOS One

Journal Requirements:

When submitting your revision, we need you to address these additional requirements.

1.Please ensure that your manuscript meets PLOS ONE's style requirements, including those for file naming. The PLOS ONE style templates can be found at

https://journals.plos.org/plosone/s/file?id=wjVg/PLOSOne_formatting_sample_main_body.pdf and

https://journals.plos.org/plosone/s/file?id=ba62/PLOSOne_formatting_sample_title_authors_affiliations.pdf

2. Please note that funding information should not appear in any section or other areas of your manuscript. We will only publish funding information present in the Funding Statement section of the online submission form. Please remove any funding-related text from the manuscript.

3. We note that you have indicated that there are restrictions to data sharing for this study. PLOS only allows data to be available upon request if there are legal or ethical restrictions on sharing data publicly. For more information on unacceptable data access restrictions, please see http://journals.plos.org/plosone/s/data-availability#loc-unacceptable-data-access-restrictions.

Before we proceed with your manuscript, please address the following prompts:

a) If there are ethical or legal restrictions on sharing a de-identified data set, please explain them in detail (e.g., data contain potentially identifying or sensitive patient information, data are owned by a third-party organization, etc.) and who has imposed them (e.g., a Research Ethics Committee or Institutional Review Board, etc.). Please also provide contact information for a data access committee, ethics committee, or other institutional body to which data requests may be sent.

b) If there are no restrictions, please upload the minimal anonymized data set necessary to replicate your study findings to a stable, public repository and provide us with the relevant URLs, DOIs, or accession numbers. For a list of recommended repositories, please see

https://journals.plos.org/plosone/s/recommended-repositories. You also have the option of uploading the data as Supporting Information files, but we would recommend depositing data directly to a data repository if possible.

We will update your Data Availability statement on your behalf to reflect the information you provide.

4. Please include your full ethics statement in the ‘Methods’ section of your manuscript file. In your statement, please include the full name of the IRB or ethics committee who approved or waived your study, as well as whether or not you obtained informed written or verbal consent. If consent was waived for your study, please include this information in your statement as well.

5. Please include captions for your Supporting Information files at the end of your manuscript, and update any in-text citations to match accordingly. Please see our Supporting Information guidelines for more information: http://journals.plos.org/plosone/s/supporting-information.

6. We note that Figure S1 includes an image of a participant  in the study.

As per the PLOS ONE policy (http://journals.plos.org/plosone/s/submission-guidelines#loc-human-subjects-research) on papers that include identifying, or potentially identifying, information, the individual(s) or parent(s)/guardian(s) must be informed of the terms of the PLOS open-access (CC-BY) license and provide specific permission for publication of these details under the terms of this license. Please download the Consent Form for Publication in a PLOS Journal (http://journals.plos.org/plosone/s/file?id=8ce6/plos-consent-form-english.pdf). The signed consent form should not be submitted with the manuscript, but should be securely filed in the individual's case notes. Please amend the methods section and ethics statement of the manuscript to explicitly state that the patient/participant has provided consent for publication: “The individual in this manuscript has given written informed consent (as outlined in PLOS consent form) to publish these case details”.

If you are unable to obtain consent from the subject of the photograph, you will need to remove the figure and any other textual identifying information or case descriptions for this individual.

7. If the reviewer comments include a recommendation to cite specific previously published works, please review and evaluate these publications to determine whether they are relevant and should be cited. There is no requirement to cite these works unless the editor has indicated otherwise.

Additional Editor Comments:

Please address the reviewers comments and address the methodological concerns or provide justification and clarity on the methodological approach and choices where necessary.

[Note: HTML markup is below. Please do not edit.]

Reviewers' comments:

Reviewer's Responses to Questions

**Comments to the Author**

1. Is the manuscript technically sound, and do the data support the conclusions?

Reviewer #1: Yes

Reviewer #2: Yes

Reviewer #3: Yes

**********

2. Has the statistical analysis been performed appropriately and rigorously?

Reviewer #1: Yes

Reviewer #2: Yes

Reviewer #3: Yes

**********

3. Have the authors made all data underlying the findings in their manuscript fully available?

The PLOS Data policy

Reviewer #1: Yes

Reviewer #2: No

Reviewer #3: Yes

**********

4. Is the manuscript presented in an intelligible fashion and written in standard English?

Reviewer #1: Yes

Reviewer #2: Yes

Reviewer #3: Yes

**********

Reviewer #1: This is an automated report for PONE-D-26-15749. This report was solicited by the PLOS One editorial team and provided by ScreenIT.

ScreenIT is an independent group of scientists developing automated tools that analyze academic papers. A set of automated tools screened your submitted manuscript and provided the report below. Each tool was created by your academic colleagues with the goal of helping authors. The tools look for factors that are important for transparency, rigor and reproducibility, and we hope that the report might help you to improve reporting in your manuscript. Within the report you will find links to more information about the items that the tools check. These links include helpful papers, websites, or videos that explain why the item is important. While our screening tools aim to improve and maintain quality standards they may, on occasion, miss nuances specific to your study type or flag something incorrectly. Each tool has limitations that are described on the ScreenIT website. The tools screen the main file for the paper; they are not able to screen supplements stored in separate files. Please note that the Academic Editor had access to these comments while making a decision on your manuscript. The Academic Editor may ask that issues flagged in this report be addressed. If you would like to provide feedback on the ScreenIT tool, please email the team at ScreenIt@bih-charite.de. If you have questions or concerns about the review process, please contact the PLOS One office at plosone@plos.org.

Reviewer #2: A) Major Revision Required: -

The manuscript addresses an important and clinically relevant topic in sports medicine by developing a field-based Clinical Triage Scoring System (CTSS) for identifying collegiate athletes requiring priority intervention for lumbopelvic-hip dysfunction. The study includes a relatively large sample (n = 864), systematic assessment procedures, and internal validation using bootstrap methods. The reported discrimination (AUROC = 0.851) and calibration appear promising.

However, several substantial methodological and reporting concerns limit confidence in the current findings. The most important issue is the potential circularity between the predictor variables and the expert-derived reference standard, which may have inflated model performance. Additional concerns relate to predictor selection strategy, outcome definition, lack of external validation, and insufficient adherence to contemporary prediction-model reporting standards (TRIPOD/TRIPOD-AI).

The manuscript has merit but requires substantial revision before it can be considered for publication.

Reviewer #3: The manuscript entitled “Development and Internal Validation of a Field-Based Triage Tool for Lumbopelvic-Hip Dysfunction in Collegiate Athletes” addresses an important topic in sports medicine and rehabilitation. The development of a practical field-based clinical triage scoring system (CTSS) for identifying athletes requiring priority intervention is relevant and may have potential clinical utility. The study includes a relatively large sample size and employs several statistical techniques, including logistic regression, ROC analysis, calibration assessment, bootstrap validation, and decision curve analysis.

The manuscript has several strengths, including a clear study objective, adequate sample size, and a clinically applicable scoring system derived from routine assessments. The reported discrimination performance (AUROC = 0.851) and calibration results indicate promising model performance.

**********

what does this mean?). If published, this will include your full peer review and any attached files.

If you choose “no”, your identity will remain anonymous but your review may still be made public.

**Do you want your identity to be public for this peer review?**  For information about this choice, including consent withdrawal, please see our Privacy Policy

Reviewer #1: No

Reviewer #2: **Yes:** Teferi G.

Reviewer #3: **Yes:** Dr. Muhammad Zahid Mahmood

**********

[NOTE: If reviewer comments were submitted as an attachment file, they will be attached to this email and accessible via the submission site. Please log into your account, locate the manuscript record, and check for the action link "View Attachments". If this link does not appear, there are no attachment files.]

To ensure your figures meet our technical requirements, please review our figure guidelines: https://journals.plos.org/plosone/s/figures

You may also use PLOS’s free figure tool, NAAS, to help you prepare publication quality figures: https://journals.plos.org/plosone/s/figures#loc-tools-for-figure-preparation.

NAAS will assess whether your figures meet our technical requirements by comparing each figure against our figure specifications.

Attachment Submitted filename: report_10.64898+2026.04.23.26351566.pdf

Attachment Submitted filename: Reviewer Report for Manuscript Number PONE-D-26-15749.docx

# Author comment: Author response to Decision Letter 1

**DOI:** 10.1371/journal.pone.0348820.r002

14 Jul 2026

Thank you for the opportunity to revise our manuscript. We sincerely appreciate the constructive comments and suggestions provided by the reviewers and editor.

We have carefully revised the manuscript accordingly, and a detailed point-by-point response to all reviewer comments is provided in the accompanying Response to Reviewers document.

In addition, we have revised the manuscript to meet PLOS ONE’s submission requirements, including updating the Supporting Information files and removing all funding-related text from the manuscript.

Following the additional editorial office request regarding potentially identifying participant information, Figure S1 has been revised by replacing the participant photograph with a non-identifiable schematic illustration of the lumbopelvic–hip assessment regions. The revised figure contains no identifiable participant information.

We hope that the revised manuscript is now suitable for consideration for publication in PLOS ONE, and we thank you for your time and consideration.

Sincerely,

Wen-Lan Wu, PhD

On behalf of all authors

Attachment Submitted filename: response-to-reviewer_ PONE-D-26-15749_revised_final3.doc

# Peer review: Decision Letter 1

**DOI:** 10.1371/journal.pone.0348820.r003

**Authors:** Aliah Shaheen

23 Aug 2026

Development and Internal Validation of a Field-Based Triage Tool for Lumbopelvic-Hip Dysfunction in Collegiate Athletes

PONE-D-26-15749R1

Dear Dr. Wu,

We’re pleased to inform you that your manuscript has been judged scientifically suitable for publication and will be formally accepted for publication once it meets all outstanding technical requirements.

Within one week, you’ll receive an e-mail detailing the required amendments. When these have been addressed, you’ll receive a formal acceptance letter and your manuscript will be scheduled for publication.

An invoice will be generated when your article is formally accepted. Please note, if your institution has a publishing partnership with PLOS and your article meets the relevant criteria, all or part of your publication costs will be covered. Please make sure your user information is up-to-date by logging into Editorial Manager at Editorial Manager® and clicking the ‘Update My Information' link at the top of the page. For questions related to billing, please contact billing support.

If your institution or institutions have a press office, please notify them about your upcoming paper to help maximize its impact. If they’ll be preparing press materials, please inform our press team as soon as possible -- no later than 48 hours after receiving the formal acceptance. Your manuscript will remain under strict press embargo until 2 pm Eastern Time on the date of publication. For more information, please contact onepress@plos.org.

Kind regards,

Aliah Faisal Shaheen

Academic Editor

PLOS One

Additional Editor Comments (optional):

Reviewers' comments:

Reviewer's Responses to Questions

**Comments to the Author**

Reviewer #3: All comments have been addressed

**********

2. Is the manuscript technically sound, and do the data support the conclusions?

Reviewer #3: Yes

**********

3. Has the statistical analysis been performed appropriately and rigorously?

Reviewer #3: Yes

**********

4. Have the authors made all data underlying the findings in their manuscript fully available?

The PLOS Data policy

Reviewer #3: Yes

**********

5. Is the manuscript presented in an intelligible fashion and written in standard English?

Reviewer #3: Yes

**********

Reviewer #3: The authors have addressed many of the concerns raised during the previous review, and the revised manuscript is substantially improved. The manuscript is generally well organized, the methodology is more clearly described, and the statistical analyses are presented in greater detail. The development of a field-based clinical triage scoring system for lumbopelvic-hip dysfunction in collegiate athletes is clinically relevant and may provide a practical tool for sports medicine professionals. The study demonstrates acceptable methodological rigor, including a relatively large sample size, internal bootstrap validation, calibration analysis, and decision curve analysis. However, the manuscript should continue to emphasize that the proposed CTSS is internally validated only. The outcome measure is based on expert panel judgment rather than an objective clinical gold standard. Although the authors justify this approach appropriately, the limitations associated with expert-derived reference standards should be discussed more explicitly, including the possibility of subjective classification bias.The authors should clarify how the CTSS can be implemented in routine sports medicine practice. Including a brief description or flowchart illustrating how clinicians, physiotherapists, athletic trainers, or sports chiropractors should apply the score in real-world field settings would improve clinical utility.

Although decision curve analysis supports the usefulness of the model, additional discussion regarding the clinical implications of false-positive and false-negative classifications would strengthen interpretation of the recommended cutoff score.

**********

what does this mean?). If published, this will include your full peer review and any attached files.

If you choose “no”, your identity will remain anonymous but your review may still be made public.

**Do you want your identity to be public for this peer review?**  For information about this choice, including consent withdrawal, please see our Privacy Policy

Reviewer #3: **Yes:** Dr. Muhammad Zahid Mahmood

**********

# Editor report: Acceptance letter

**DOI:** 10.1371/journal.pone.0348820.r004

**Authors:** Aliah Shaheen

PONE-D-26-15749R1

PLOS One

Dear Dr. Wu,

I'm pleased to inform you that your manuscript has been deemed suitable for publication in PLOS One. Congratulations! Your manuscript is now being handed over to our production team.

At this stage, our production department will prepare your paper for publication. This includes ensuring the following:

* All references, tables, and figures are properly cited

* All relevant supporting information is included in the manuscript submission,

* There are no issues that prevent the paper from being properly typeset

You will receive further instructions from the production team, including instructions on how to review your proof when it is ready. Please keep in mind that we are working through a large volume of accepted articles, so please give us a few days to review your paper and let you know the next and final steps.

Lastly, if your institution or institutions have a press office, please let them know about your upcoming paper now to help maximize its impact. If they'll be preparing press materials, please inform our press team within the next 48 hours. Your manuscript will remain under strict press embargo until 2 pm Eastern Time on the date of publication. For more information, please contact onepress@plos.org.

You will receive an invoice from PLOS for your publication fee after your manuscript has reached the completed accept phase. If you receive an email requesting payment before acceptance or for any other service, this may be a phishing scheme. Learn how to identify phishing emails and protect your accounts at https://explore.plos.org/phishing.

If we can help with anything else, please email us at customercare@plos.org.

Thank you for submitting your work to PLOS One and supporting open access.

Kind regards,

PLOS One Editorial Office Staff

on behalf of

Dr. Aliah Faisal Shaheen

Academic Editor

PLOS One
